# Seagrass (*Posidonia oceanica*) seedlings in a high-CO_2_ world: from physiology to herbivory

**DOI:** 10.1038/srep38017

**Published:** 2016-12-01

**Authors:** Gema Hernán, Laura Ramajo, Lorena Basso, Antonio Delgado, Jorge Terrados, Carlos M. Duarte, Fiona Tomas

**Affiliations:** 1 Departament of Ecology and Marine Resources, Mediterranean institute for advanced studies (CSIC-UIB), 07190, Esporles, Balearic Islands, Spain; 2 Departament of Global Change Research, Mediterranean institute for advanced studies (CSIC-UIB), 07190, Esporles, Balearic Islands, Spain; 3 Department of Science, Liberal Arts School. Universidad Adolfo Ibáñez, 2640, Santiago, Chile; 4 Center of Research and Innovation for Climate Change (CiiCC). Universidad Santo Tomás, Santiago, Chile; 5 Department of Biological and Environmental Sciences and Technologies (DiSTeBA), University of Salento, 73100 Lecce, Italy; 6 Stable Isotope Biogeochemistry Laboratory, Andalusian Institute of Earth Science (CSIC-UGR), 18100 Armilla, Granada, Spain; 7 King Abdullah University of Science and Technology (KAUST), Red Sea Research Center (RSRC), Thuwal, 23955-6900, Saudi Arabia; 8 Department of Fisheries and Wildlife, Oregon State University, Corvallis, OR, USA

## Abstract

Under future increased CO_2_ concentrations, seagrasses are predicted to perform better as a result of increased photosynthesis, but the effects in carbon balance and growth are unclear and remain unexplored for early life stages such as seedlings, which allow plant dispersal and provide the potential for adaptation under changing environmental conditions. Furthermore, the outcome of the concomitant biochemical changes in plant-herbivore interactions has been poorly studied, yet may have important implications in plant communities. In this study we determined the effects of experimental exposure to current and future predicted CO_2_ concentrations on the physiology, size and defense strategies against herbivory in the earliest life stage of the Mediterranean seagrass *Posidonia oceanica*. The photosynthetic performance of seedlings, assessed by fluorescence, improved under increased *p*CO_2_ conditions after 60 days, although these differences disappeared after 90 days. Furthermore, these plants exhibited bigger seeds and higher carbon storage in belowground tissues, having thus more resources to tolerate and recover from stressors. Of the several herbivory resistance traits measured, plants under high *p*CO_2_ conditions had a lower leaf N content but higher sucrose. These seedlings were preferred by herbivorous sea urchins in feeding trials, which could potentially counteract some of the positive effects observed.

Seagrass meadows are of utmost importance for marine coastal systems. These photosynthetic organisms form the basis of diverse coastal food webs having important ecological functions and offering crucial services for humans^1^. As seagrasses are highly productive, they act as a carbon sink^2^,^3^,^4^ and, as ecosystem engineers, they provide substratum for epiphytic plants and animals to settle^5^ and support coastal and offshore fisheries^6^. However, seagrasses are presently suffering a global loss associated to numerous human activities^7^,^8^.

Early life stages of plants play a crucial role in providing a new genetic variation pool necessary for adaptation to future environmental changes^9^,^10^,^11^. In seagrasses this is a critical life stage since, in some species, sexual reproduction is very variable in space and time and in general, there is a very low rate of seedling establishment^12^. Although some seagrass species rely mainly on clonal reproduction, sexual reproduction provides genetic diversity which could make seagrass populations more resilient and resistant to future changes^13^. Furthermore, seeds and seedlings provide a key opportunity to colonize new habitats^14^, and under the current scenarios of anthropogenic threats, this strategy may be particularly relevant for allowing the establishment of seagrass into more suitable environments.

Atmospheric CO_2_ concentrations have increased between 1.3% and 3.4% y^−1^ over the last decade^15^,^16^, and projections based on current emission rates estimate that atmospheric concentrations would increase up to 800 ppm by the end of the century^17^ and up to 1500–2100 ppm between 2100 and 2200^18^,^19^. Plants with C3 metabolism are predicted to benefit by this increase in CO_2_ concentrations in two ways. First, increasing CO_2_ availability increases carbon fixation rates^20^,^21^. Secondly, a higher *p*CO_2_ will reduce photorespiration because of the higher diffusion of CO_2_, increasing the efficiency of carbon uptake^22^,^23^ and overall photosynthesis^24^. As a result of the increased carbon assimilation, studies in terrestrial plants show increases in biomass, non-structural carbohydrates and C:N ratios^25^. Due to the absorbing potential of the ocean (30–40% of anthropogenic CO_2_ released to the atmosphere^26^), seawater would increase its *p*CO_2_, with more CO_2_ available for photosynthetic organisms. Since most seagrasses have a C3 photosynthetic metabolism, they are predicted to benefit by the increase in CO_2_ availability^21^. Indeed, even though most seagrass species have carbon-concentrating mechanisms, they seem to be limited by the current CO_2_ concentration, as increased CO_2_ availability in the water enhances photosynthesis^27^,^28^,^29^. However, how this predicted increase in photosynthesis would translate into increase in plant performance or abundance remains unclear although long term experiments and studies in CO_2_ vents found increased productivity and density respectively at low pH (7.6 and 7.3)^30^,^31^. Besides, there are no conclusive patterns about long-term effects of elevated *p*CO_2_ in carbon budget or chemical composition in most seagrasses^32^,^33^,^34^,^35^.

Furthermore, beyond plant productivity effects, changes in CO_2_ concentrations may also modify the capacity of plants to resist or tolerate herbivory^36^. Plants have developed diverse mechanisms against herbivory; their tolerance strategies reduce the impact of herbivory on plant fitness (e.g. increasing carbon storage to regrow after damage) and their resistance strategies reduce the feeding preference or performance of the herbivore (e.g. decreasing nutritional quality)^36^,^37^. Indeed, plant nutritional quality as well as chemical defenses are key resistance traits in controlling plant consumption by herbivores^37^,^38^. Increased *p*CO_2_ often decreases nutritional quality (increasing C/N^35^,^39^) and increases chemical defenses (e.g. phenolic compounds) in terrestrial plants, with consequences for plant-herbivore interactions^40^,^41^. Nevertheless, the scarce available literature in seagrasses suggest a decrease, rather than an increase, in the concentration of phenolic compounds with increased *p*CO_2_^42^,^43^. Furthermore, the consequences of CO_2_-driven changes in seagrass defense strategies, as well as the consequences for plant-herbivore interactions remain poorly studied, and so far no clear general patterns emerge^43^,^44^,^45^,^46^.

In addition, environmental changes in resource availability, such as high CO_2_ concentrations, may affect the trade-offs in resource allocation between growth and secondary metabolism in plants^47^,^48^ with significant ecological costs (e.g. outcome of interactions with herbivores, pathogens or competitors) that might be difficult to predict *a priori*. Understanding the effects of elevated *p*CO_2_ on the performance of seagrass seedlings is important as they represent a particularly vulnerable period experiencing high mortality rates^49^. Furthermore, herbivore pressure exerted on seedlings has critical effects on plant populations^50^, shaping composition and structure of plant communities^51^. Because of the critical ecological importance of early life stages and the likely physiological differences with adult stages, the effects of future high *p*CO_2_ in plant early life stages require specific examination.

In this study we hypothesized that under increased CO_2_ availability seagrass seedlings 1) would increase their incorporation of carbon and perform better and therefore, 2) become less palatable for herbivores and thus, less preferred. To test this, we experimentally assessed the effects of increased CO_2_ concentrations predicted for the end of 21st century in newly emerged seedlings of *P. oceanica* on several morphological and physiological responses. Additionally, we estimated the effects of high *p*CO_2_ on seedling survivorship and their palatability to herbivores. To our knowledge, this is the first study examining the effects of increased CO_2_ availability on early life stages of seagrasses and the implications for interactions with herbivores. Given the importance of early life history changes in providing potential for adaptation and colonization to new environments, understanding how increased CO_2_ will influence the performance of seagrass seedlings is critical for evaluating the consequences of future CO_2_ increases on seagrass populations.

## Results

### Seedling photosynthetic traits

ETRmax and saturation irradiance (Ek) were significantly higher (27% and 24% respectively) after 60 days of treatment in the leaves of seedlings grown under high *p*CO_2_ (Supplementary material Fig. S1, Table 1). While there were no differences in AF (control *p*CO_2_: 0.641 ± 0.025, high *p*CO_2_: 0.628 ± 0.08, one-way ANOVA: F_(1/12)_ = 0.023, P = 0.89), maximum quantum yield (Y) nor in the photosynthetic quantum efficiency (α) between *p*CO_2_ treatments (Table 1). Furthermore, no differences in photosynthetic parameters were evident after 90 days between treatments, with ETRmax and Ek decreasing in both treatments (Supplementary material Fig. S1, Table 1).

### Seedling size and mortality

**Figure 1:**
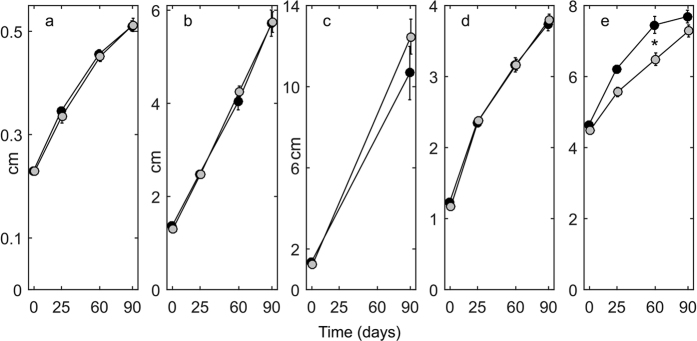
Leaf width (**a**), maximum leaf length (**b**), root length (**c**), number of roots (**d**), and number of leaves (**e**) measured in seedlings growing under high CO_2_ (black) or control (grey) conditions, at the beginning of the experiment and after 25,60 and 90 days. Total root length (**c**) was only measured at the beginning and after 90 days of experiment. Error bars indicate standard error. Asterisk indicates statistically significant differences between treatments.

**Figure 2:**
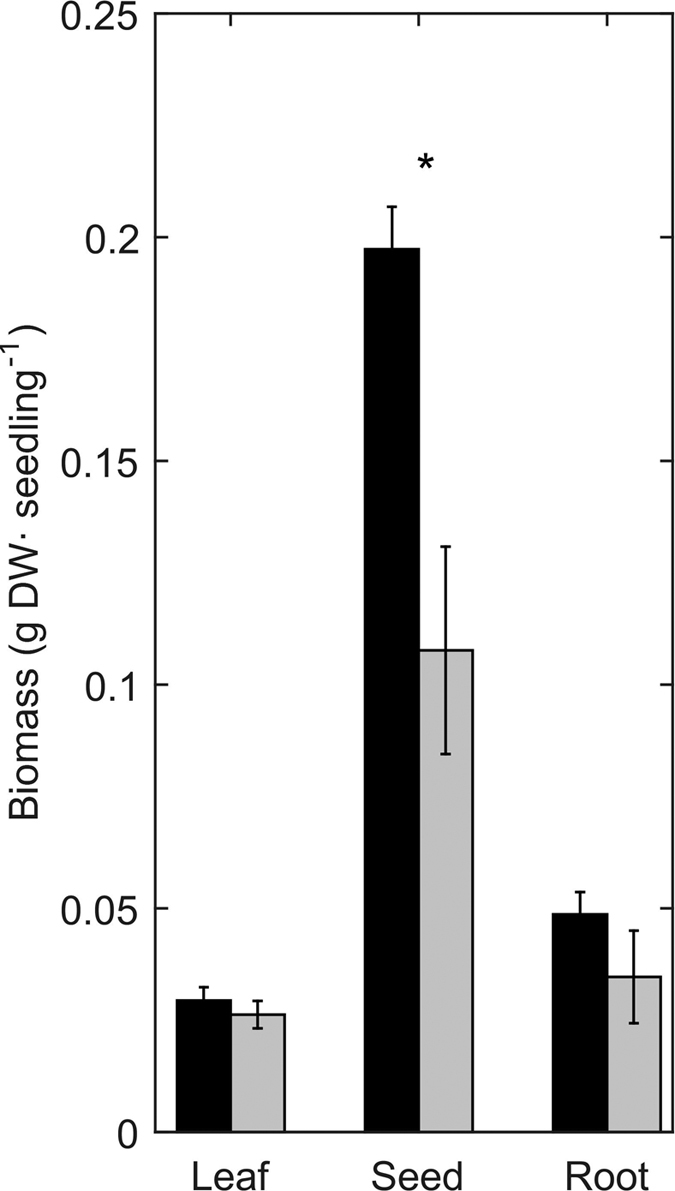
Mean dry Biomass (g) of leaves, seeds and roots of seedlings growing under high CO_2_ (black) or control (grey) conditions. Error bars indicate standard error. Asterisk indicates statistically significant differences between treatments.

While initial seedling size (i.e. leaf width, maximum leaf length, number of leaves, number of roots, and total root length) was similar between treatments, the number of leaves was significantly higher in seedlings from the high *p*CO_2_ treatment after 60 days (Fig. 1, Table 2). Leaf width, maximum leaf length and number of roots did not differ between treatments despite substantial growth along the experimental period. No significant difference between *p*CO_2_ treatments was found in total root length after 90 days (Fig. 1, Table 1). CO_2_ concentrations did not affect the mortality of seedlings (control *p*CO_2_: 7.6 ± 2.79%, high *p*CO_2_: 8.4 ± 3.1%, one-way ANOVA: F_(1/12)_ = 0.04, P = 0.84). Seed biomass under high *p*CO_2_ was almost 2-fold higher than for control seedlings, while there where no differences between treatments for leaf and root biomass (Fig. 2, Table 3).

### Seedling chemical traits

The δ13C in leaves of seedlings from the high *pCO*_*2*_ treatment (−20.5 ± 1.19 δ‰) was significantly lower than in control plants (−16.4 ± 1.39 δ‰), (Table 3).

**Figure 3:**
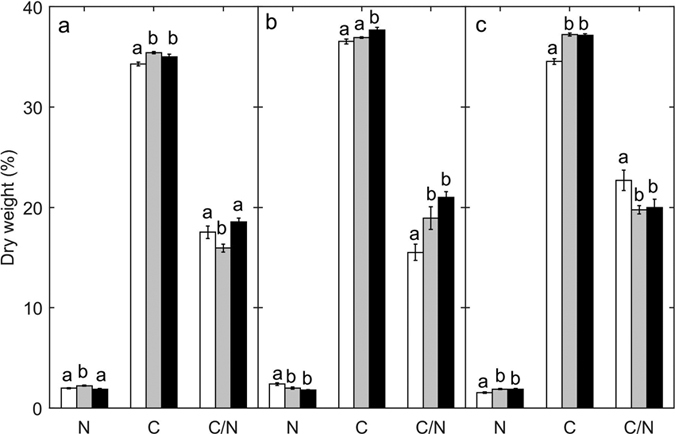
Percentage of dry weight in Nitrogen (N), Carbon (C), and Carbon Nitrogen ratio (C/N), in leaves (**a**), seeds (**b**) and roots (**c**) of seedlings at the beginning of the experiment (white) or after growing under high CO_2_ (black) or control (grey) conditions. Error bars indicate standard error and different letters indicate statistically significant differences across treatments (Tukey).

**Figure 4:**
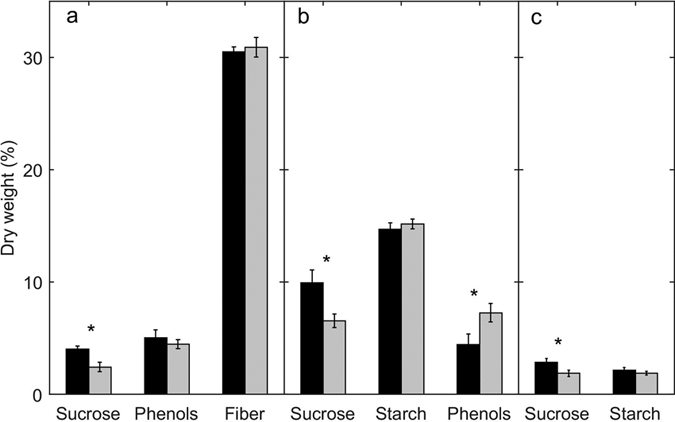
Percentage of dry weight in sucrose, starch, total phenol content (Phenols), and fiber in leaves (**a**). seeds (**b**) and roots (**c**) of seedlings growing under high CO_2_ (black) or control (grey) conditions. Error bars indicate standard error. Asterisk indicates statistically significant differences between treatments.

Carbon content increased in leaves and roots when compared to the beginning of the experiment, but differences related to CO_2_ treatments were only observed in seeds, which had a higher C content under high *p*CO_2_ (Fig. 3 Table 3). The nitrogen content of seeds decreased and that of roots increased throughout the experiment and did not differ between experimental treatments. Conversely, leaf nitrogen content was ca. 17% lower in the high *p*CO_2_ when compared to the control, which increased by 13% throughout the experimental period. Hence, the leaf C/N ratio was 14% higher in CO_2_-enriched plants when compared to controls (Fig. 3, Table 3). CO_2_ enrichment resulted in higher (more than 30%) content of sucrose in seeds and roots, while no significant changes were detected between treatments in starch content. Sucrose content in leaves was almost two fold higher in the increased *p*CO_2_ treatment compared to the non-enriched (Fig. 4, Table 3). The increase in CO_2_ availability did not affect the total phenolic content (Kruskal Wallis test. χ2 = 0.102, df = 1, P = 0.749) nor the fiber content (Fig. 4, Table 3) of leaves, while it significantly decreased (39%) phenolic content in the seeds (Kruskal Wallis test, χ2 = 6.208, df = 1, P = 0.013).

### Herbivore feeding experiment

**Figure 5:**
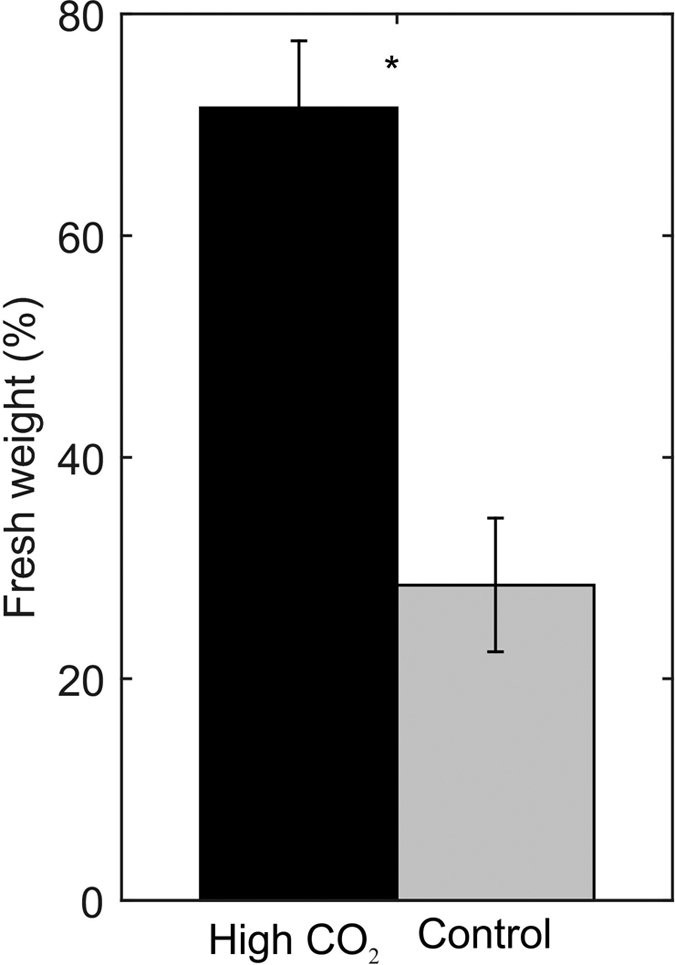
Percentage of fresh weight consumed by herbivores from leaf tissue of seedlings growing under high CO_2_ (black) or control (grey) conditions. Error bars indicate standard error. Asterisk indicates statistically significant differences between treatments.

Sea urchins consumed a significantly higher amount of fresh leaf tissue biomass from leaves grown under high *p*CO_2_ in comparison to control *p*CO_2_ conditions (Wilcoxon signed-ranks paired test, z = 2.78, n = 19, P = 0.004, Fig. 5).

## Discussion

Early life seagrass stages could benefit under the future elevated CO_2_ predicted scenarios. Our results show that, in general, seedling photosynthetic performance was enhanced under elevated *p*CO_2_ levels during the initial phases of seedling development, leading to increased sucrose content of leaves, roots and seeds, and an overall increase in carbon storage. These positive effects could translate into having more resources stored to resist or recover from stressful conditions. On the other hand, increased CO_2_ availability led to biochemical changes in leaves that resulted in shifts in the palatability of this tissue.

While currently the major source of photosynthetic inorganic carbon uptake in *P. oceanica* seems to be in form of HCO_3_^−^ rather than CO_2[aq],_ a future increase in CO_2[aq]_ may change this ratio^28^,^52^. The higher ETRmax observed after 60 days of experiment suggests a greater ability to transfer electrons under high CO_2_ conditions. Interestingly, seeds of *Posidonia* spp seedlings have photosynthetic activity that enhances seedling growth^53^. While we did not measure the photosynthetic activity of the seed, a higher CO_2_ availability could have also increased photosynthesis in this organ, potentially contributing to a higher total photosynthetic activity when compared to seedlings from other species or adults.

As demonstrated by the δ^13^C values in our study, seedlings from high *p*CO_2_ treatments exhibited reduced CO_2_ fractionation, suggesting that seedlings growing under present CO_2_ are likely CO_2_-limited. In addition, leaves from the increased *p*CO_2_ treatment had a higher content of sucrose, an effect that has been also found in other studies with adult seagrasses^39^. Sucrose is the principal end-product of leaf photosynthesis^54^; and the higher content found in our study is thus likely resulting from the increased photosynthetic activity during the early development of the seedlings. In general, higher CO_2_ availability increases photosynthetic activity in seagrasses^30^,^34^,^35^,^55^ which sometimes translates into increases in aboveground biomass or growth^33^,^35^,^39^. However this increase in photosynthesis and thus in carbon incorporation is not always allocated to aboveground growth^32^,^34^,^45^. In our study, seedlings under high *p*CO_2_ did not allocate carbon to changes in aboveground size at the end of the experimental period but rather to maintain or slow the decrease of seed biomass. Similarly, adult seagrasses can also exhibit an increase in belowground biomass^56^ or changes in the chemical composition of below and aboveground tissues^32^,^45^.

In this study, seeds exhibited lower sucrose content in the control treatment, which suggests that seedlings growing under high *p*CO_2_ had a lower consumption of sucrose from seeds or that sucrose was produced through photosynthesis, mobilized to belowground tissues and stored in seeds. This effect of increased non-structural carbohydrates in belowground tissues has been also found in adult seagrasses under experimental increase in CO_2_ availability^32^,^39^. In seedlings, this is particularly important since seeds store and supply carbon and nutrients to the seedling during the first year of its life^57^. Increased carbon reserves and biomass of the seed would benefit seedling survival and resilience to stressful conditions, especially in seagrasses such as *P. oceanica* in which the buoyant fruits disperse to new habitats away from the original meadow. Having more resources to tolerate or resist adverse light and temperature conditions or damage by herbivores would likely improve seedling establishment and survival^58^, which are key features of a successful population expansion process. Particularly in *P. oceanica* in which flowering frequency varies greatly spatially and among years (0–26%)^59^ with a low reproductive success (3–11% of seedlings available for establishment) mainly due to seed predation^60^.

Despite the cost of less carbon available for growth, carbon allocation to defense and storage often results in higher survivorship of organs and individuals^61^. Secondary metabolites are associated with defense mechanisms in plants (e.g. feeding deterrence^62^,^63^) being for some herbivores more determinant of their preference than other attributes such as carbohydrates or fibers^64^. According to the resource availability hypothesis (RAH^65^) plants grown under high resource availability will invest less in defense components than plants grown under limited resourced environments. Therefore, seagrasses grown under elevated nutrient availability (usually a limiting resource^66^) often decrease the production of chemical defenses such as phenols^67^,^68^. Being carbon-based compounds, most of the studies in terrestrial plants^25^ and some species of macroalgae^69^ have found increases in phenolic compounds with elevated CO_2_ availability. Yet, because CO_2_ is also a resource that can greatly limit primary production in seagrasses^28^,^52^,^70^, we may expect a decrease in phenolics (rather than the increase often observed in terrestrial plants) under high CO_2_ scenarios, following RAH. Indeed both a decrease^42^,^43^ as well as no changes^45^, but never an increase, in phenolic compounds have been reported in seagrass leaves growing under elevated CO_2_ conditions. While we did not find significant changes in phenol content in leaves associated with CO_2_ availability, we did observe it in seeds. Since defense has a cost, not all plant parts are equally defended, as they contribute differently to fitness^71^,^72^. Seeds have multiple important functions (e.g. carbon storage, nutrient supply and photosynthesis) which are critical for seedling survival. Thus, seeds may be an organ whose defense is prioritized under resource-limited conditions (e.g. present-day levels of CO_2_). Seeds from seedlings of the control treatments had significantly higher phenol content than those from the increased *p*CO_2_ treatment, which were bigger (higher biomass), with higher carbon content and more stored sucrose. Having more resources (i.e. CO_2_) available in the environment may have decreased the investment of carbon on seed defense towards favoring the storage of other more rapidly available carbon-based compounds such as sucrose^73^.

A decrease in nutritional quality (as a decrease in nitrogen or increased C/N content) in response to high CO_2_ has been commonly observed in terrestrial^25^,^74^ and marine plants^32^,^35^,^39^, and it has been attributed to a dilution of nitrogen due to increased leaf growth^75^, increases in leaf carbohydrates and structural material, higher plant internal nitrogen requirements^76^,^77^ and/or reductions in protein concentrations^78^. Some studies in *Zostera noltei* also found a lower N content under high *p*CO_2_ conditions together with a lower nitrate uptake^79^. This reduced nitrate uptake could be the reason for the lower nitrogen content observed in leaves in the high *p*CO_2_ treatment in this study, which would not be related to a dilution of nitrogen by increased growth since there were no differences in leaf biomass at the end of the experiment. The reduced nutritional quality observed in seagrass leaves could have consequences for herbivores that may compensate this low nutritional quality by increasing their feeding rates^80^,^81^.

Unexpectedly, in our study, leaves with lower nitrogen content were preferred by sea urchins, whereas herbivores typically prefer tissues with higher N content^82^,^83^,^84^. However, N content also includes nitrogen in insoluble forms and alkaloids^84^, and does not necessarily reflect availability and quality for herbivores. In addition, factors other than nitrogen content may also be influencing the palatability of seagrass to herbivores.

Leaf fiber content may reduce the preference of grazers by reducing the digestibility to herbivores^85^, increasing leaf toughness^86^ or decreasing the preference for high carbon-fiber plant species^87^. The neutral detergent fiber method measures most of the groups of structural constituents of plant cells (e.g. cellulose, lignin, hemicellulose). Yet, not all the components are similar in terms of production costs and defensive properties. Lignin provides better structural and chemical defensive properties than cellulose, which has half the biosynthesis cost in glucose equivalents^88^. Therefore, even though we did not detect differences in the fiber content between treatments, we cannot rule out that the relative composition of chemical components of the fiber could have differed under high CO_2_ concentrations^43^,^89^,^90^, and consequently, may have modified the palatability of the tissues.

One of the biochemical traits that changed with higher *p*CO_2_ availability was sucrose content in leaves, which may have enhanced plant palatability. In insects, for instance, sugars increase stimulation to taste^91^ and can mask the deterrent effect of other compounds^92^. Additionally, we performed the feeding experiments only with the sea urchin *P. lividus*, whereas different herbivore species may have responded differently to CO_2_-driven changes in plant chemical composition^45^,^74^ or epiphyte abundance or composition since it is expected that fleshy epiphytes may increase^93^ and calcareous epibionts would decrease their abundance under low pH conditions^30^. While we only performed the feeding experiments under ambient CO_2_ water conditions, studies to date with adult sea urchins do not suggest strong changes in feeding rates^94^,^95^, nor in preferences (S.R. Fitzpatrick, personal communication) under ambient vs. high *p*CO_2_ conditions.

In summary, the results of our experiment suggest that seedlings of *P. oceanica* might perform better under a high CO_2_ scenario. The enhanced photosynthetic activity and carbon fixation increased the amount of resources available for storage, which would benefit these early life stages to resist or recover from stress. Yet, positive effects might be counterbalanced by changes in grazing pressure due to increased palatability, although allocation of resources to tolerance could allow seedlings to survive and persist to shifts in herbivory pressure.

## Materials and Methods

### Fruit collection and seed germination

Beach-stranded fruits of *Posidonia oceanica* were collected in Palma Bay (Mallorca, Balearic Islands, Western Mediterranean) during May 2013 and transported to the laboratory in a cooler with seawater. Seeds were extracted from the fruits and maintained in aquaria at constant temperature (17 °C) with UV-filtered seawater for approximately one month until the initiation of the experiment.

### Experimental design and setup

To evaluate the effect of CO_2_ availability on *P. oceanica* seedlings, seawater was aerated with a mix of air and pure CO_2_ gasses using Mass Flow Controllers (Aalborg, USA) in order to obtain experimental CO_2_ values of actual (ca.500 ppm hereafter control treatment) and future oceanic conditions (ca. 1550 ppm, hereafter high *p*CO_2_ treatments). Seventeen seedlings of homogeneous size (control *p*CO_2_: 0.759 ± 0.007 g wet mass seedling^−1^, high *p*CO_2_: 0.758 ± 0.010 g wet mass seedling^−1^; one-way ANOVA: F_(1/12)_ = 0.015; P = 0.908) were randomly assigned to each of the seven replicate 9-L aquaria with control or high *p*CO_2_ treatments and maintained in these conditions for 90 days in 14:10 h (light: dark) light cycle. In order to maintain pH conditions and to avoid changes in other parameters of carbonate systems (i.e. alkalinity) seedlings were grown without substrate. Aquariums were cleaned and re-filled every 7 days with filtered seawater (10 μm plus UV filter) and CO_2_ pre-treated seawater to maintain stable salinity levels and water quality.

### Water conditions

Two discrete pH samples (total scale) were taken once a week from each aquarium and analyzed by spectrophotometric method under controlled temperature (17 °C). At the same time, two replicate water samples (50 cc) from each aquarium were taken for dissolved inorganic carbon (DIC) and Total Alkalinity (A_T_). Water samples were fixed with supersaturated HgCl_2_ (Merck, Analar) to avoid biological activity and changes in A_T_ conditions. A_T_ values were obtained by double endpoint titration to pH 4.45 and 4.41 (NBS scale) with HCl (Fixanal^®^) according to Dickson Sop 3b (version 3.01), using a Tritando 808 and Aquatrode plus (Metrohm^®^). The accuracy of measurements was checked against certified reference seawater (CRM, Batch 101, Dickson Scripps Institution of Oceanography, San Diego, USA). Salinity was measured daily (Hanna Instruments) and maintained at 36 psu while light and temperature were continuously recorded using HOBO data loggers (Onset^®^). Carbonate system parameters were estimated using CO2SYS^96^ with dissociation constants (K1 and K2) according to Millero *et al*.^97^ and KHSO_4_ dissociation constant after Dickson^98^. A summary of experimental treatment conditions is shown in Supplementary material Table S1.

### Seedling photosynthetic traits

Photosynthetic measurements were performed by pulse amplitude modulated (PAM) fluorometry (Walz, Effeltrich, Germany) on the seedlings after 60 and 90 days of exposition to control and high *p*CO_2_ conditions. First, the maximum quantum yield on dark-adapted seedlings was determined in three seedlings per aquarium by applying a saturating light pulse in the second leaf of each seedling after a 5-min period of dark-adaptation. To reduce variability within seedlings, all measurements were made approximately 2 cm above the leaf meristem. Effective quantum yield was measured after 10 s-exposures to 0, 11, 36, 72, 82, 140, 231, 300 and 455 μmol m^−2^ s^−1^ photon flux densities to obtain Rapid Light Curves (RLCs) in the same dark-adapted seedlings. Leaf absorbance (AF) was measured by placing 1–4 layers of leaves in front of the PAR sensor instrument and recording the percentage light absorbed by the seagrass^99^. AF was calculated as 1- exp (-α) where α is the slope of the linear correlation of the ln of the light transmitted against the number of leaf layers. Electron Transport Rates (ETR) from the RLC data were calculated as ETR = yield × irradiance × 0.5 × AF^100^. ETR values were plotted against the incident absorbed PAR and the photosynthetic quantum efficiency (α) was calculated as the slope of the linear part of the light response curve and the saturation irradiance (Ek) as the division of ETRmax by the initial slope. The maximum electron transport rate (ETRmax) and the maximum quantum yield (Y) were calculated as the maximum ETR and effective quantum yield (∆F/Fm’) of each ETR-PAR curve.

### Seedling size and mortality

Leaf width of the second leaf, maximum leaf length, total root length and number of leaves and roots of each seedling were measured at the beginning of the experiment and after 25, 60 and 90 days with the exception of root length that was only measured at the beginning and at the end of the experiment (90 days) to avoid damage. Leaf thickness was measured in the second leaf at the mid-point of their length with a precision caliper (resolution 0.01 mm) in three seedlings per aquaria. Seedling mortality was calculated as the percentage of seedlings dead after 90 days relative to the initial number of seedlings placed in each replicate aquarium. A seedling was considered dead when all leaves were shed from the sheath or necrotic. After 90 days, five seedlings were randomly selected from each experimental aquarium and dried for 48 h at 60 °C to determine biomass of leaves, roots and seed of each one.

### Seedling chemical traits

Effects of CO_2_ enriched seawater in the inorganic carbon intake of leaves, were analyzed using stable isotope ratios. Leaves of four seedlings per aquaria were dried (60 °C for 48 hours), ground and treated with HCL fumes (37%, 12–24 h) to remove carbonates^101^. Stable isotopes signatures were analyzed from 0.5 mg in a NC1500 elemental analyzer (Carlo Erba, Milan, Italy) combined with a Delta Plus XL isotope ratio mass spectrometer (ThermoQuest, Bremen, Germany). Isotope ratios in samples were calculated as:


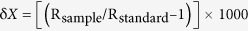


where X is ^13^C, and R is the corresponding ratio of ^13^C/^12^C.

Commercial CO_2_ was used as working standard and two internal standards with δ^13^C −30.63‰ and −11.65‰ (Vienna- PDB, V-PDB) were used for the isotopic analyses. For carbon, 22 internal standards (organic and inorganic material) ranging from −49.44‰ to + 28.59‰ (V-PDB) were contrasted with the IAEA international references NBS-28, NBS-29, NBS-20 (carbonates) and NBS-22, IAEA-CH-7, IAEA-CH-6 (organic material). The precision, calculated after correction of the mass spectrometer daily drift, was ± 0.1‰ for δ^13^C.

Regarding seedling traits related to herbivory, we considered total phenolic compounds, fiber, nitrogen content (% dry weight, DW), sucrose content (%DW) and C/N of leaves as resistance traits, whereas the number of leaves, and the carbon (% DW), sucrose and starch (%DW) content of seeds and roots were considered tolerance traits.

Pooled plant material (ca. 6 seedlings) of each experimental aquarium was ultrafrozen (−80 °C), freeze-dried, and ground to a fine powder to determine the concentration of carbon, nitrogen, fiber, sucrose and total phenols in leaves and carbon, nitrogen, starch and sucrose in seeds and roots and total phenols in seeds. In order to have an initial reference on carbon and nitrogen contents, 6 samples of seedlings (pooled plant material of ca. 6 seedlings each) were freeze-dried at the beginning of the experiment.

Carbon and nitrogen content in leaves, seeds and roots were analyzed using a Carlo-Erba CNH elemental analyzer (EA1108). Total phenols were extracted from ca. 4 mg of ground tissue with 1.5 mL of methanol 50% for 24 h and were determined with spectrophotometer (Hitachi, U-2900) following a modified Folin-Ciocalteu method using caffeic acid as standard (modified from Bolser *et al*.^102^). Non-structural carbohydrates in leaves (sucrose), and seeds and roots (sucrose and starch) were measured using methodology described by Invers^66^. Sucrose and other soluble sugars were obtained after three sequential extractions with 95% (v/v) ethanol at 80 °C for 15 min. The remaining pellet of roots and seeds was dissolved in 0.1N NaOH for 24 h at room temperature for starch extraction. Soluble sugars and starch contents of extracts were determined by spectrophotometry using an anthrone assay with sucrose as standard. Neutral detergent fiber content (NDF) was measured in 25–30 mg of leaf sample (see de los Santos *et al*.^103^, modification from Van Soest *et al*.^104^). The amount of NDF in each sample was obtained by difference in dry biomass and is referred as ‘fiber content’ hereafter.

### Feeding experiment

In order to examine how biochemical changes due to increased CO_2_ availability modify plant palatability, we performed a feeding assay with herbivorous sea urchins. After 90 days of treatment, four seedlings from each experimental aquarium of both *p*CO_2_ treatments were used in a two-choice experiment. The feeding assay was performed in an indoor seawater flow-through system (i.e. ambient CO_2_ conditions) and a light:dark photoperiod of 12:12 h. Similar-sized sea urchins (4.98 ± 0.66 cm, one-way ANOVA: F_(1/22)_ = 1.695; P = 0.21) of the species *Paracentrotus lividus*, the main invertebrate herbivore on *P. oceanica* meadows, were acclimated for a period of 48 hours and fed with *Ulva lactuca* ad libitum. Individual urchins were placed in cages of 225 cm^2^ covered with a 1 cm mesh and offered similar amounts of leaf tissue clean of epiphytes (c.a. 3–4 leaves from one seedling) from control and high CO_2_ treatments. Control cages without herbivores were used to measure any potential changes in leaf tissue not related to grazing. The weight changes in the controls were used to correct the autogenic changes in the feeding replicates. The corrected consumption was calculated as:


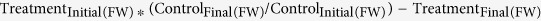


The experiment consisted of 20 replicates and ended when approximately 50% of initial material was consumed. Following the procedures of previous feeding behavior experiments^45^, replicates in which all the offered samples were either totally consumed or fully intact were not considered in the statistical analysis.

### Statistical analyses

Differences in initial wet weights between treatments as well as seedling mortality and size of sea urchins were analyzed using a one-way ANOVA analysis. Plant size traits were analyzed using repeated measures ANOVA analyses with time (days) as within-subject factor and *p*CO_2_ treatment (high and control) as the between subject factor. The effects of experimental treatments on plant chemical traits (leaf total phenol content, leaf thickness, leaf fiber content, non-structural carbohydrates of leaves, seeds and roots, and biomass of leaves, seed and roots) obtained at the end of the experiment were analyzed by means of one-way ANOVA tests. Carbon and nitrogen contents of leaf, seed and roots were also compared with the initial samples with a one-way ANOVA. Total phenolic content of leaves and seeds was analyzed with Kruskal-Wallis rank sum test as the data were not normal even after transformation. The mean value of each aquarium was used as replicate for all the above-mentioned analyses. The analysis of two-choice experiments was performed using a Wilcoxon signed-ranks paired test. *Post hoc* analyses were performed with Tukey multiple comparisons of means. Data were checked for normality with the Saphiro-Wilk test and homogeneity of variances with the Bartlett test. ANOVAs were conducted without transformation of the variables.

## Additional Information

**How to cite this article**: Hernán, G. *et al*. Seagrass (*Posidonia oceanica*) seedlings in a high-CO_2_ world: from physiology to herbivory. *Sci. Rep.* **6**, 38017; doi: 10.1038/srep38017 (2016).

**Publisher's note:** Springer Nature remains neutral with regard to jurisdictional claims in published maps and institutional affiliations.

## Supplementary Material

Supplementary Information

## Figures and Tables

**Table 1:** Mean, standard error and results of repeated measures ANOVA in photosynthetic parameters and Tukey HSD test (H: HighCO_2_, C: Control CO_2_, 60: 60 days 90: 90 days, n = 7).

Variable	Control CO_2_		High CO_2_		Source	DF	DFerror	MS	MSerror	F	P	Tukey’s tests
	60 days	90days	60 days	90days								
Maximum Quantum Yield	0.708 (0.012)	0.682 (0.179)	0.712 (0.008)	0.705 (0.008)	CO_2_	1	12	0.00	0.00	1.10	0.314	
					Time	1	12	0.00	0.00	2.28	0.157	
					Time x CO_2_	1	12	0.00	0.00	0.74	0.406	
Alfa	0.179 (0.007)	0.144 (0.009)	0.186 (0.006)	0.15 (0.008)	CO_2_	1	12	0.00	0.00	0.61	0.452	
					Time	1	12	0.01	0.00	27.32	**<0.001**	90 < 60
					Time x CO_2_	1	12	0.00	0.00	0.02	0.899	
Maximum electron transport rate (ETRmax)	12.15 (0.92)	9.11 (0.462)	16.73 (0.99)	9.11 (0.419)	CO_2_	1	12	36.66	3.69	9.93	**0.008**	C < H
					Time	1	12	198.68	4.11	48.31	**<0.001**	90 < 60
					Time x CO_2_	1	12	36.60	4.11	8.90	**<0.001**	C90 = H90 < C60 < H60
Saturation Irradiance (Ek)	68.35 (4.68)	64.34 (3.56	90.65 (5.9)	62.91 (3.03)	CO_2_	1	12	762.10	197.00	3.87	0.0728	
					Time	1	12	1764.60	79.40	22.23	**<0.001**	90 < 60
					Time x CO_2_	1	12	985.70	79.40	12.41	**0.004**	C90 = H90 < C60 < H60

**Table 2:** Results of Repeated Measures ANOVA in morphometric parameters, and Tukey HSD test (H: HighCO_2_; C: Control CO_2_; 0: beginning experiment; 25: 25 days; 60: 60 days; 90: 90 days, n = 7).

Variable	Source	DF	DFerror	MS	MSerror	F	P	Tukey’s tests
Leaf Width	CO_2_	1	12	0	0.001	0.12	0.733	
	Time	3	36	0.219	0	514.37	**<0.0001**	0 < 25 < 60 < 90
	Time x CO_2_	3	36	0	0	0.18	0.909	
Maximum Leaf Length	CO_2_	1	12	0.034	0.333	0.1	0.754	
	Time	3	36	51.38	0.1	525.88	**<0.0001**	0 < 25 < 60 < 90
	Time x CO_2_	3	36	0.05	0.1	0.54	0.661	
Number of Leaves	CO_2_	1	12	4.153	0.31	13.4	**0.003**	C60 < H60
	Time	3	36	23.39	0.126	184.99	**<0.0001**	0 < 25 < 60 < 90
	Time x CO_2_	3	36	0.426	0.126	3.29	**0.032**	C60 < H60
Number of Roots	CO_2_	1	12	0.001	0.055	0.02	0.923	
	Time	3	36	17.31	0.029	602.5	**<0.0001**	0 < 25 < 60 < 90
	Time x CO_2_	3	36	0.009	0.029	0.32	0.811	
Total Root Length	CO_2_	1	6	0.8	3.99	0.18	0.674	
	Time	3	23	740.3	4.5	162.9	**<0.0001**	0 < 90
	Time x CO_2_	3	23	6.5	4.5	1.43	0.244	

**Table 3:** Results of one way ANOVAs in plant traits and Tukey HSD test (I: Initial, H: HighCO_2_, C: Control CO_2_, n = 7).

Tissue	Plant Traits	DF	DFerror	MS	MSerror	F	P	Tukey’s tests
Leaf	C (%DW)	2	17	2.115	0.235	8.995	**0.002**	I < H = C
	N (%DW)	2	17	0.218	0.023	9.591	**0.002**	I = H < C
	C/N	2	17	12.09	1.37	8.824	**0.002**	C < H = I
	Sucrose (%DW)	1	12	9.072	0.848	10.7	**0.007**	C < H
	Fiber (%DW)	1	12	0.582	3.306	0.18	0.682	
	Thickness (cm)	1	12	0	0	0.031	0.863	
	Biomass (g)	2	12	0	0	0.27	0.768	
	δ13 C	1	12	59.49	1.047	56.83	**<0.001**	H < C
Seed	C (%DW)	2	17	2.285	0.264	8.673	**0.002**	I = C < H
	N (%DW)	2	17	0.569	0.072	7.875	**0.004**	H = C < I
	C/N	2	17	49.24	5.22	9.429	**0.002**	I < C = H
	Sucrose (%DW)	1	12	40.42	5.83	6.94	**0.022**	C < H
	Starch (%DW)	1	12	0.682	1.711	0.398	0.54	
	Biomass (g)	2	12	0.015	0.02	6.837	**0.01**	C < H
Root	C (%DW)	2	17	14.71	0.24	61.19	**<0.001**	I < H = C
	N (%DW)	2	17	0.25	0.028	8.957	**0.002**	I < H = C
	C/N	2	17	17.034	3.975	4.286	**0.031**	H = C < I
	Sucrose (%DW)	1	12	3.324	0.626	5.31	**0.04**	C < H
	Starch (%DW)	1	12	0.214	0.388	0.55	0.472	
	Biomass (g)	2	12	0	0	0.794	0.474	
