# Supplementary material for: Seagrass (Posidonia oceanica) seedlings in a high-CO2 world: from physiology to herbivory
Source: Sci Rep. 2016 Dec 1;6:38017. doi: 10.1038/srep38017 (PMC5131316; doi:10.1038/srep38017)
Supplement: Supplementary Information [file srep38017-s1.pdf]

Title: Seagrass (*Posidonia oceanica*) seedlings in a high-CO_2 world: from physiology to herbivory.

Running head: Performance of seagrass seedlings under high CO_2 availability.

List of authors

Gema Hernán[1], Laura Ramajo[2,3,4], Lorena Basso[2,5], Antonio Delgado[6], Jorge Terrados[1], Carlos M. Duarte[2,7] and Fiona Tomas[1,8].

Affiliations

[1]Departamento de Ecología y Recursos marinos IMEDEA (CSIC-UIB), Instituto Mediterráneo de Estudios Avanzados, C/ Miquel Marqués 21, 07190 Esporles, Islas Baleares, Spain

[2]Departamento de Cambio Global, IMEDEA (CSIC-UIB), Instituto Mediterráneo de Estudios Avanzados, C/ Miquel Marqués 21, 07190 Esporles, Islas Baleares, Spain

[3]Departmento de Ciencias. Facultad de Artes Liberales. Universidad Adolfo Ibáñez, Avda. Diagonal Las Torres 2640, Santiago, Chile.

[4]Centro de Investigación e Innovación para el Cambio Climático (CiiCC). Universidad Santo Tomás, Ejercito 146. Santiago, Chile

[5]Department of Biological and Environmental Sciences and Technologies (DiSTeBA), University of Salento, 73100 Lecce, Italy.

[6]Laboratorio de Biogeoquímica de Isótopos Estables. Instituto Andaluz de Ciencias de la Tierra, IACT (CSIC-UGR), 18100 Armilla, Granada

[7]King Abdullah University of Science and Technology (KAUST), Red Sea Research Center (RSRC), Thuwal, 23955-6900, Saudi Arabia

[8]Department of Fisheries and Wildlife, Oregon State University, Corvallis, OR, US.

Corresponding author: Gema Hernan. E-mail: ghernan@imedea.uib-csic.es

Supplementary Material

Table S1. **Experimental conditions**. Average (± SE) water conditions and carbonate system parameters of experimental aquaria (with seedlings, n = 7) and reference aquarium (without seedlings, n = 1) during incubations of seedlings of *P. oceanica* exposed to control and high CO_2 conditions for 90 days.

**Variable**	**Control CO_2**	**High CO_2**
Temperature (ºC)	16.36 ± 0.53	16.19 ± 0.75
Light (µmol s^{-1} m^{-2})	71.07 ± 5.32	73.59 ± 5.69
pH_T (17ºC)	8.06 ± 0.02	7.61 ± 0.02
A_T (mmol/kgSW)	2984 ± 29	2971 ± 40
pCO_2 (µatm)	535 ± 21	1574 ± 47
$[CO_3^{2-}]$ (mmol/kgSW)	233 ± 7	97 ± 5
$[HCO_3^{-}]$ (mmol/kgSW)	2337 ± 41	2743 ± 29
$\Omega_{aragonite}$	3.54 ± 0.11	1.47 ± 0.07
$\Omega_{calcite}$	5.48 ± 0.17	2.27 ± 0.10

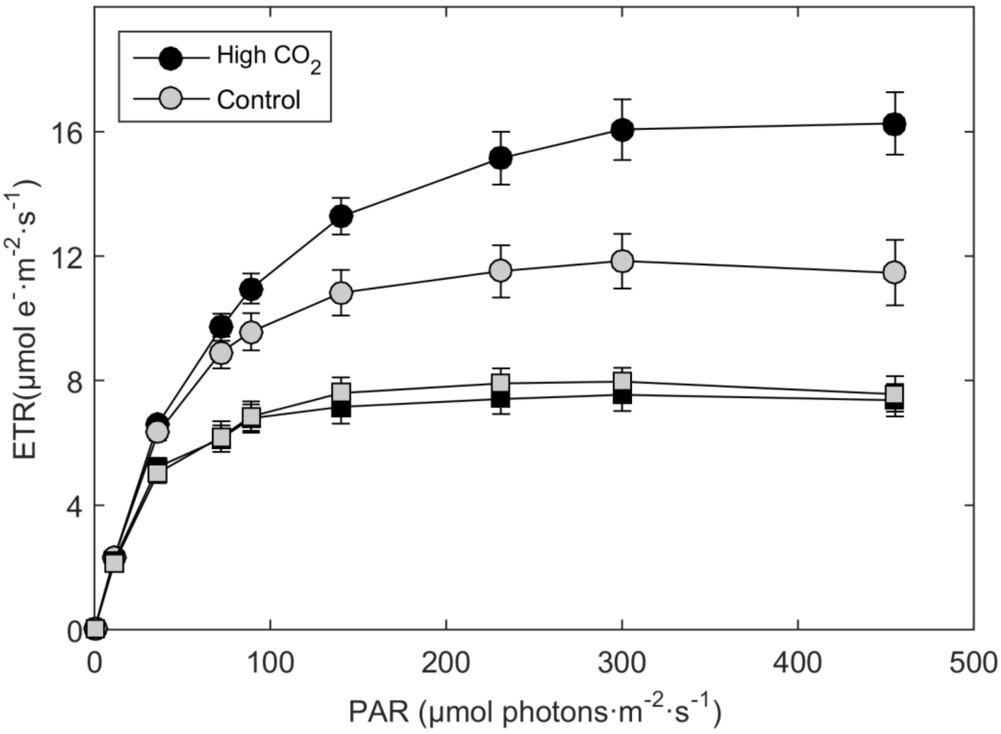

Figure S1. ETR-light curves. Electron transport rate estimated from Rapid Light Curves performed in seedlings growing under high CO_2 (black) or control (grey) conditions after 60 (circles) and 90 (squares) days of experiment. Error bars indicate standard error.
